# The Multifaceted Role of Platelets in Atherosclerosis and Ischemic Disease: Pathogenesis, Inflammation, and Therapeutic Opportunities

**DOI:** 10.3390/life15111656

**Published:** 2025-10-23

**Authors:** Sophia Strukel, Betelhem Teshome, Vikrant Rai

**Affiliations:** 1 College of Osteopathic Medicine of the Pacific, Western University of Health Sciences, Pomona, CA 91766, USA; sophia.strukel@westernu.edu; 2 Department of Translational Research, Western University of Health Sciences, Pomona, CA 91766, USA; bteshome@westernu.edu

**Keywords:** atherosclerosis, plaque rupture, chronic inflammation, platelets, therapeutics

## Abstract

(1) Background: Atherosclerosis is a complex chronic inflammatory disease characterized by the plaque-induced thickening of medium-sized and large arterial walls. Chronic inflammation, lipid accumulation, and endothelial dysfunction play a critical role in pathophysiology of atherosclerosis. Along with immune cells, vascular smooth muscle cells, endothelial cells, and platelets play a critical role in the pathogenesis of atherosclerosis. Targeting platelet-related molecular mechanisms has emerged as a promising therapeutic strategy in treating atherosclerosis. However, potential targets are not clearly understood. This review discusses the multifaceted role of platelets in the pathogenesis of atherosclerosis and ischemic disease followed by the potential of targeting platelets. (2) Methods: Articles related to the role of platelets in atherosclerosis and underlying molecular mechanisms were searched from PubMed and Google Scholar using search terms atherosclerosis, platelets, therapeutics, targets; alone or in combination; (3) Results: Current research suggest that platelet-related molecular mechanisms play a critical role in plaque development, progression, and rupture. The mediators involved may serve as therapeutic targets; (4) Conclusions: Targeting platelets can attenuate atherosclerosis by interfering with platelet functions beyond blood clotting, such as promoting vascular inflammation and platelet adhesion.

## 1. Introduction

Atherosclerosis is a complex disease, characterized by the plaque-induced thickening of medium-sized and large arterial walls [1,2]. It is a chronic inflammatory disorder, resulting from the accumulation of fatty acids, cholesterol, calcium, fibrin, cellular debris, and waste products in the vascular subendothelium. This buildup leads to the formation of a nascent fatty streak within the arterial intima, followed by atheroma and atheromatous plaque, which may eventually evolve into a fibrous plaque, causing varying degrees of arterial stenosis and impairing organ perfusion [1]. Plaque progression involves inflammatory mediators, including toll-like receptors, triggering receptors expressed on myeloid cell-1, cytokines, alarmins, macrophages, dendritic cells, collagen degradation, and transcription factors regulating various mediators and molecular mechanisms [3,4,5]. As plaques enlarge, they become unstable and prone to rupture. Unstable plaques are characterized by increased inflammation, necrotic core, decreased vascular smooth muscle cell proliferation, and a thin plaque cap. Plaque rupture can trigger blood coagulation and thrombosis at the rupture site, further occluding local or downstream vessels. In severe cases, this may result in complete occlusion, causing myocardial infarction (MI), stroke, or peripheral artery disease (PAD) [1,6].

The concept of atherosclerosis as an inflammatory disease was suggested by Russel Ross in 1999, based on observations of circulating monocytes infiltrating developing fatty streaks. However, the specific antigens involved in the initiation of this inflammation remain under investigation [7]. Genome-wide association, clonal linear tracing, and clinical trials have revealed that both innate and adaptive immune systems exert activating and inhibitory effects on atherosclerosis-mediated inflammation [6,7,8]. Autoantigens, such as oxidized low-density lipoprotein (ox-LDL) and antigens released from apoptotic cells within plaques, have been shown to promote plaque formation, impair apoptotic clearance, and intensify localized inflammation [2,7].

Recent studies have further examined the relationship between lipids and inflammation, providing a complementary perspective to the traditional “lipid hypothesis”. In addition to the lipid-driven risk, a metabolic–immune model has been proposed in which pro-inflammatory lipid effects stimulate vascular hyperplasia, even in the absence of traditional cardiovascular risk factors [7]. Mauricio et al. [9] emphasize that atherosclerosis is a multifactorial disease and that the lipid and immune–metabolic hypotheses should be viewed not as mutually exclusive but rather, as closely interconnected.

Since atherosclerosis has been an asymptomatic condition for many years, its true incidence is difficult to determine and estimated based on cardiac events rather than initial disease process. However, it remains the primary cause of many cardiovascular diseases (CVDs), including heart failure, PAD, coronary artery disease (CAD), and strokes [10]. Furthermore, atherosclerosis is the underlying cause of nearly 50% of all deaths in Western society [6]. Approximately 75% of acute MIs are caused by plaque ruptures. Given that CVDs are the leading cause of mortality worldwide, and that atherosclerosis is a central driver of this burden, understanding its pathophysiology is crucial for designing therapies that effectively reduce its risk [11].

Among the key contributors to the pathophysiology of atherosclerosis are platelets. These small anucleate cells classically function to form plugs at the site of vascular injury, preventing bleeding. Once activated, platelets aggregate and release bioactive molecules that help strengthen their plug. However, in pathological conditions such as atherosclerosis, their role can extend far beyond hemostasis. Platelets contribute to angiogenesis, secrete proinflammatory chemokines and cytokines, and alter the vascular microenvironment to promote further plaque formation. For example, they upregulate adhesion molecule expression on endothelial cells (ECs), facilitating leukocyte recruitment. This promotes foam cell development, a hallmark of atherosclerosis, as macrophages engulf low-density lipoprotein (LDL) particles and form the core of atherosclerotic plaques [10]. Platelets also directly contribute to the formation of a weak, necrotic plaque core, predisposing the fibrous cap to rupture and subsequent thrombus formation [10,12].

**Figure 1:**
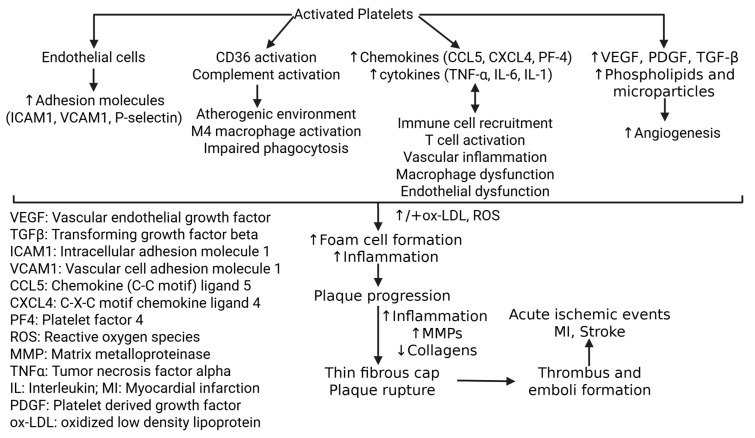
Mechanisms of platelet-mediated plaque formation and rupture. Increased adhesion molecules, CD36 and complement activation, increased production of chemokines and cytokines, increased immune cell recruitment contributing to chronic inflammation, and growth factors promoting angiogenesis contribute to plaque formation. Persistent inflammation and matrix degeneration results in plaque rupture, thrombus formation, and acute ischemic events. ↑ indicates increased expression and ↓ indicates decreased expression.

Although platelets are primarily categorized as hemostatic cells, they are now recognized as active players in inflammation and vascular pathology [13]. They release chemokines and cytokines, such as chemokine C-C motif ligand 5 (CCL5), also known as RANTES (regulated upon activation normal T expressed and secreted), and platelet factor 4 (PF-4), that contribute directly to immune cell recruitment and vascular inflammation. For example, when thrombin stimulates human platelets, they release a preformed protein with eosinophil chemotactic activity that can be purified to form RANTES, a chemokine that selectively recruits monocytes and T cells [13,14]. In vivo murine experiments also demonstrated that injection of activated platelets exacerbated atherosclerosis by P-selectin-dependent deposition of PF-4 and RANTES [15]. Platelets also release other pro-inflammatory cytokines such as tumor necrosis factor (TNF)-α, which is a potential biomarker for predicting plaque rupture and whose inhibition has been shown to reduce plaque progression and vascular inflammation [16,17] (Figure 1).

Beyond their inflammatory activity, platelets act as powerful regulators of vascular remodeling and angiogenesis. Upon activation, platelets release two proteins essential for angiogenesis, including platelet-derived growth factor (PDGF) and transforming growth factor beta (TGF-β) [10]. Platelets are also a rich source of circulating vascular endothelial growth factor (VEGF), a pro-angiogenic growth factor that can act in the intravascular membrane of atherosclerotic vessels [18] (Figure 1). In conjunction with the release of cytokines, upon sensing damage, platelets also release platelet-derived phospholipids and microparticles that work as synergistic regulators of angiogenesis [19]. Interestingly, Tersteeg et al. [20] demonstrated platelet-induced remodeling of the atherosclerotic vascular environment by showing that activated platelets will expose long negatively charged membrane strands called flow-induced protrusions (FLIPRs), which can capture circulating monocytes and neutrophils to promote the formation of platelet–leukocyte microparticle complexes and platelet microparticles (PMPs), further inducing inflammation and foam cell formation.

This review will examine the multifaceted role of platelets in the pathogenesis of atherosclerosis and ischemic disease, with an emphasis on their contributions beyond traditional hemostasis. We will explore mechanistic insights into how platelet-driven processes, including inflammation, immune modulation, angiogenesis, and plaque destabilization, intersect with vascular pathology. In addition, we will highlight emerging therapeutic opportunities that target platelet-mediated pathways, aiming to reduce both the burden of thrombosis itself and the chronic inflammatory environment that sustains atherosclerosis. By understanding the platelet pathophysiology behind atherosclerosis from a clinical application viewpoint, this review seeks to provide a comprehensive understanding of platelets as both drivers of disease and potential targets for innovative treatment strategies.

## 2. Platelets in the Initiation and Progression of Atherosclerosis

### 2.1. Early Involvement in Lesion Formation

**Figure 2:**
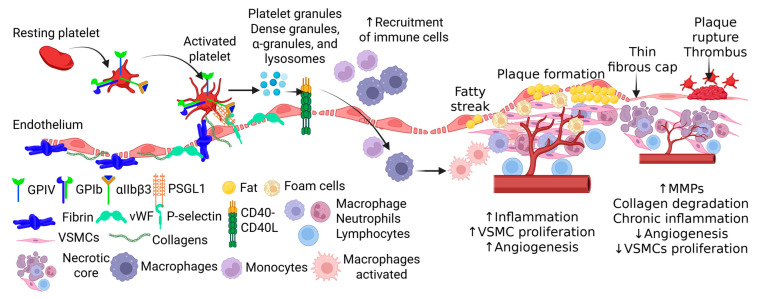
Plaque formation and rupture involving platelets. Activated platelets secrete platelet granules which in turn activate CD40-CD40L interaction. This increases immune cell recruitment in the vessel contributes to plaque formation. This also increases foam cell formation, vascular smooth muscle cell (VSMCs) proliferation, angiogenesis contributing to plaque progression. Persistent inflammation and immune cells, oxidative stress, and collagen degradation results in thin fibrous cap, the point of plaque rupture. ↑ indicates increased expression and ↓ indicates decreased expression.

Platelets are localized to vascular lesions by recognizing exposed extracellular matrix (ECM) proteins such as von Willebrand factor (vWF) and collagen. They bind to vWF through the glycoprotein Ib//IX/V (GPIb/IX/V) receptor and to collagen via GPVI. These interactions initiate platelet activation and induce conformational changes in integrins, particularly αIIbβ3 (GPIIb/IIIa, fibrinogen receptor) and α2β1 (collagen receptor), allowing for firm adhesion of their respective ECM ligands (Figure 2). Subsequently, activated platelets then aggregate, forming a platform for fibrin bridges that further strengthen the developing thrombus [21]. In addition to ECM binding, platelets can transiently interact with vascular ECs through a process called platelet rolling. This mechanism involves an initial loose connection between platelets and ECs, mediated by selectins. During this process, platelet surface receptors GPIbα and P-selectin glycoprotein ligand-1 (PSGL-1) interact with P-selectin expressed on the activated endothelium. However, the interactions between P-selectin and PSGL-1 or GPIb/IX/Vis are rapidly reversible and insufficient for stable adhesion [21] (Figure 2).

As adhesion proceeds, platelets undergo activation and release a wide array of inflammatory and pro-atherosclerotic mediators into the local environment. These mediators are packaged in three major platelet granule types: dense granules, α-granules, and lysosomes. In circulating platelets, granules remain sequestered until activation triggers their exocytosis [22]. Upon release, the granules and micro-vesicles upregulate monocyte proinflammatory surface markers such as CD40 and procoagulant tissue factor (TF), a principal initiator in coagulation. Additionally, monocytes exposed to platelet secretions of TNF-α, monocyte chemoattractant protein-1 (MCP-1), and interleukin 1β (IL-1β) adopt proinflammatory transcriptomes (Figure 2). Furthermore, platelets skew monocyte and macrophage differentiation toward a pro-atherosclerotic phenotype [23]. Additional cargo released from platelet granules includes P-selectin, fibrinogen, vWF, VEGF, and chemokines such as C-X-C motif chemokine ligand 1 (CXCL1) and interleukin (IL)-8, which act synergistically to promote adhesion, aggregation, chemotaxis, proliferation, coagulation, and proteolysis [22]. Together, these factors amplify inflammatory processes and drive immune cell recruitment.

### 2.2. Immune Cell Interaction and Inflammation

Atherosclerosis-compounded endothelial dysfunction results from impairments in the preventative mechanisms established to prevent platelet activation [24]. Inadequacies in the inner blood vessel lining’s ability to regulate blood flow and inflammation result in endothelial activation and further promotes a pro-inflammatory environment. Additionally, reduced nitric oxide (NO) availability, increased oxidative stress, and chronic disorders, such as hypercholesterolemia, insulin resistance, hyperglycemia, and smoking, can all trigger hemodynamic flow dysregulation [24]. Activated endothelium recruits platelets, which tether to them and subsequently become activated [24].

Platelets play an essential role in recruiting inflammatory cells to sites of vascular injury. Activated platelets release chemokines that create a strong chemotactic gradient, guiding monocytes toward the endothelium [25]. Once at the vessel wall, monocytes interact with platelets to form platelet–monocyte complexes (PMCs) through P-selectin-PSGL-1 interactions (Figure 2). These complexes enhance monocyte adhesion to the endothelium and increase their capacity for trans-endothelial migration, with studies showing up to a threefold increase in translocation when compared to free monocytes. Following transmigration, monocytes dissociate from PMCs. Here, macrophages and monocytes play a prominent role in cholesterol accumulation, matrix remodeling, cytokine production, and clearance of apoptotic cells [26]. Notably, Martins et al. reported that monocytes bound to platelets exhibit a higher activation state and increased atherogenic capacity [27].

Beyond facilitating chemotaxis and transmigration of monocytes, platelets also shape the phenotypes of infiltrating immune cells. C-X-C motif chemokine ligand 4 (CXCL4), one of the most abundant chemokines released from α-granules, drives monocyte differentiation into proinflammatory M4-type macrophages, which play a crucial role in plaque rupture [22] (Figure 1). Additionally, in vitro murine models of atherosclerosis have shown that ox-LDL generated under chronic inflammatory conditions enhances platelet–monocyte interactions, promoting monocyte extravasation and foam cell formation [28]. The ox-LDL-activated platelets bind to proinflammatory monocytes through the P-selectin-PSGL1 axis and form an envelope around the PMCs. EC uptake of ox-LDL promotes the formation of foam cells and PMCs transmigration across the endothelium. PMCs splay a role in proinflammatory signals cascading into coagulation, thrombus formation, and plaque vulnerability- all key characteristics of atherosclerotic lesions [24]. Additionally, microparticles released in the presence of ROS become biochemical markers for cellular stress and intercellular inflammatory cascades [23]. These biochemical signals play a crucial role in endothelial dysfunction and immune response. A positive feedback mechanism further activates platelets to promote ox-LDL formation, which insidiously progresses to form foam cells, inflammation, and plaque aggregation [28,29].

Platelets also interact with neutrophils, forming neutrophil–platelet aggregates (NPAs) [23]. Platelets stimulate neutrophils to release neutrophil-extracellular traps (NETs) into circulation. These structures contain DNA, histones, and enzymes such as myeloperoxidase, which further contribute to localized thrombosis and vascular inflammation [23,30,31]. The interaction of NETs with lipids and inflammatory cytokines, along with the activation of platelets and other immune cells, creates a damaging environment that initiates and fuels atherosclerotic lesion development. NETs acting in concert with lipids and other inflammatory mediators to promote a pro-inflammatory environment within the arterial wall, activating platelets, recruiting inflammatory cells, and contributing to the destabilization and potential rupture of atherosclerotic plaques, ultimately contributing to disease progression, risk of thrombosis, and acute events [32,33].

### 2.3. Release of Inflammatory Factors

An important component of the atherosclerosis–platelet interface is the myriad of chemokines, cytokines, and growth factors present. As atherosclerotic plaques develop, CXCL4 is internalized through the blood vessel walls where it functionally alters vascular smooth muscle cell (VSMC) proliferation, migration, gene expression and cytokine release [34]. CXCL4 serves as one of the most abundant, bi-influential chemokines in platelet-derived α-granules, inhibiting LDL binding and degradation, while encouraging LDL-induced intimal tearing and other pro-atherogenic modifications. Kaczor et al. elaborate on CXCL4’s contributive effects toward VSMC-derived foam cells, crediting the chemokine’s enhanced binding of oxLDL to human VSMCs [34]. The duality in VSMC–platelet interactions becomes less ambiguous when the stage of plaque formation is detailed clearly. Though effects of CXCL4 might appear one-dimensional, as the upregulation of oxLDL advances plaque progression, this chemokine executes contextually dependent, regulatory roles guided by the inflammatory environment [24].

Murine models of atherosclerosis have demonstrated that CXCL4 deficiencies reduce atherosclerotic lesion formation. Overall, platelet secretion of CXCL4 has been shown to enhance harmful lipoprotein aggregation and further advance atherosclerosis development. Another notable chemokine in the atherosclerotic environment is CCL5 (RANTES). In atherosclerotic arteries, activated platelets induce monocytes to release CCL5-containing microparticles. CCL5 then acts on CCR5 and C-C chemokine receptor type 1 (CCR1) receptors [35]. CCL5 deposition then enhances monocyte recruitment by inflammatory aortic ECs, which allows it to serve as a biomarker for chronic CAD and atherosclerosis initiation.

CXCL4 and CCL5 also interact to form heterodimers that draw monocytes to inflammatory, vascular ECs and drive the production of plaque aggregation and NET formation. Disruption of the heterodimer using MKEY peptide inhibitors (an inhibitor of the CXCL4-CCL5 heterodimer) leads to attenuated leukocyte recruitment, reduced NET formation, and a significant decrease in infarct size [35]. Nording et al. affirms the inhibition of the functional heterodimer and elaborates upon the CXCL4-CCR5 interaction’s ability to reduce inflammation and atherosclerosis, without impairing immune function [36].

In response to activation, ECs release chemokines, cytokines, and adhesion molecules, resulting in various paracrine interactions amongst neighboring cells and other blood components [35]. Chemokines, cytokines, and growth factors collectively shape the proinflammatory environment within the artery wall. The expression of inflammatory receptors and cytokines that recruit leukocytes reflects the defensive reaction to cardiovascular risk described by Wang et al. [35]. Atherosclerotic lesions tend to localize at the arterial curvatures or bifurcations where low blood flow and shear stress exert mechanical strain on ECs; this hemodynamic stress on vascular ECs activates inflammatory pathways, including oxidative stress and cytokine-mediated leukocyte recruitment [24].

In contrast to chemokines, which mediate targeted cell responses, cytokines encompass a broader range of signaling proteins secreted by immune cells during inflammation. Microbial infections or cellular damage detected by a functional inflammasome trigger inflammation and cleave dormant proteins, activating inflammatory cytokines [37]. Cytokine IL-1β induces endothelial activation and enhances the expression of adhesion molecules, vascular cell adhesion molecule-1 (VCAM-1) and intercellular adhesion molecule 1 (ICAM-1), thereby strengthening EC adhesion. Beyond reinforcing cell adhesion, IL-1β promotes proinflammatory production of chemokines and cytokines, such as IL-6 [37].

Ma et al. [38] developed a biomimetic therapeutic platform to mitigate atherosclerotic progression through targeted immunomodulation. The researchers highlight IL-6 and TNF-α as proinflammatory cytokines directly involved in inflammation, while IL-10 and TGF-β are recognized as anti-inflammatory mediators serving protective roles. The comprehensive study depicts significant progression in translational atherosclerotic research as it demonstrates the therapeutic potential of targeted modulation of inflammatory pathways cascading into atherosclerotic lesions. Further discussion will explore the biochemical insights and clinical implications of this approach.

### 2.4. Monocyte-to-Foam Cell Transformation

Following recruitment, monocytes differentiate into macrophages and begin to accumulate lipids forming foam cells. Platelets promote this transformation by enhancing the uptake of ox-LDL by monocytes and macrophages, which simultaneously inhibits monocyte apoptosis and stimulates cell migration to atherosclerotic lesions [39]. Through the release of CXCL4, platelets induce differentiation into M4 macrophages, a proinflammatory phenotype with the impaired ability to phagocytose apoptotic cells [40]. This reduced ability to clear apoptotic cells promotes inflammation and contributes to the expansion of the necrotic core, destabilizing the plaque [39,41] (Figure 1). However, M4 macrophages are still able to engulf ox-LDLs leading to the accumulation of lipids and eventual transition to foam cells. Foam cells not only lose efferocytotic capacity but also undergo multiple forms of programmed cell death, including apoptosis, autophagy, necroptosis, and pyroptosis [8]. The accumulation of dead and dying foam cells further enlarges the necrotic core and weakens fibrous cap integrity, resulting in increased risk of plaque rupture [42].

Furthermore, platelets directly interact with ox-LDL to amplify vascular pathology. Ligation of platelet CD36 by ox-LDL induces rapid changes in platelet shape and promotes platelet hyperreactivity, driving a pro-atherogenic environment [43]. Murine models have also demonstrated that CD36 overexpression accelerates thrombosis, lipid accumulation, foam cell formation, endothelial apoptosis, and vascular inflammation [43,44]. Interestingly, platelets reciprocally contribute to LDL oxidation by enhancing the production of ROS and low-density lipoprotein receptor (LDLR) degradation via the formation of ox-LDL proprotein convertase subtilisin/kexin type 9 [45]. Overall, platelet dysfunction and hyperlipidemia work congruently to aggravate dyslipidemia, platelet activation, and atherosclerotic progression.

### 2.5. Platelet-Derived Microparticles

Extracellular vesicles (EVs) are membranous subcellular structures classified according to morphology, size, molecular content, cellular origin, and function [46,47]. Structurally, EVs are enclosed by a lipid bilayer, which contains particles that can be produced by a wide range of cells [48]. Distinguished by their biogenetic pathways, release mechanisms, and physiology, EVs have three primary subtypes: exosomes, microvesicles, and apoptotic bodies [47]. These particles act as messengers as they carry their molecular cargo from one cell to another, influencing how cells behave. Exosomes, enveloped in an outer membrane, are vesicles that derive from inward budding of early endosomes’ limiting membranes and develop into multivesicular bodies (MVBs) [47,49]. Exosomes are present in various kinds of body fluids and can be seen engaging in endocytosis and material trafficking that play roles in protein sorting, recycling, storage, transport, and release [47,50]. Originally believed to be cellular dump sites, microvesicles develop from outward budding and play a part in intercellular communication between cells [51]. Apoptotic bodies are generated when cellular contraction increases the hydrostatic pressure and, in turn, forces the cell membrane to detach from the cytoskeleton [47]. Dying cells are then discharged as apoptotic bodies into the extracellular environment [52]. However, unlike exosomes and microvesicles, apoptotic bodies include intact organelles, chromatin, and glycosylated proteins [47].

The pathophysiological roles of EVs are influenced by the spatial arrangements between the proteins, nucleic acids, and lipids within the vesicle. Functional outcomes of EVs, like inflammation, cell communication, coagulation, and metastasis, can be modified by the phenotype and physiological state of the recipient cell. Platelet-derived extracellular vesicles are released during platelet activation and serve as the most common type of EVs circulating in plasma [47]. Due to the abundance of these EVs that are released when the endothelium is disrupted, platelet microparticles (PMPs) have operated as core drivers in atherosclerosis and thrombosis [37].

Platelet activation initiates intercellular communication between ECs and immune cells through secreted factors, ligand receptors, and PMPs [37]. The interaction of ox-LDL with leukocytes and ECs is what triggers the vascular activation and chemokine secretion, propagating leukocyte recruitment, adhesion, and transmigration [28]. The complexes formed by activated platelets and circulating leukocytes serve as a significant marker for cardiovascular risks. Leukocytes, especially monocytes and neutrophils, are recruited through the activated endothelium as they differentiate into the macrophages that engulf lipids present within the plaque [38]. The progression of foam cells drives lipid storage within the plaque, promoting lipid-rich plaques with foam cells enveloped within a fibrous, smooth muscle cell cap. The significance of foam cells lies in their ability to act as the earliest hallmark for atherosclerotic lesions [38].

### 2.6. Complement Activation and Amplification

Platelets actively engage the complement system, a key component of innate immunity, to shape the genesis and progression of atherosclerosis [53]. Upon activation, platelets promote P-selectin-mediated activation of C3, leading to the release of C3a, an anaphylatoxin and potent inflammatory mediator. Complement activation further drives assembly of C5b-9 membrane attack complexes (MACs) on platelet surfaces, which enhance platelet activation and procoagulant activity [54]. This establishes a feed-forward loop in which platelet activation triggers complement deposition (P-selectin mediated C3b) and complement activation (C3 and C5) in turn amplifies platelet activity, sustaining thrombo-inflammatory processes within the plaque microenvironment. Platelet-mediated complement activation is a physiological process where activated platelets trigger the complement system, an innate immune defense, to generate inflammatory mediators, like C3a and C5a, and promote cell clearance. This interaction plays a role in vascular injury, thrombosis, and autoimmune diseases like immune thrombocytopenia [55]. Complement activation plays a dual role in atherosclerosis, both promoting and potentially protecting against the disease depending on the context and pathway involved. While the classic and lectin pathways can clear apoptotic cells, pro-inflammatory products from alternative pathway activation, such as anaphylatoxins and the terminal complement complex, contribute to endothelial activation, immune cell recruitment, and plaque destabilization, increasing the risk of cardiovascular events [56,57].

Complement activity varies by plaque depth. In the luminal layer of atherosclerotic lesions, both classical and alternative pathways are detectable, although terminal complement activation is suppressed, likely due to regulation by complement inhibitors such as C4bp and factor H. In contrast, MAC deposition is prominent in deeper regions of the intima, where it is associated with smooth muscle cells, extracellular lipids, and necrotic debris [58]. Because of their high susceptibility to complement-mediated activation, platelets express a broad repertoire of complement control proteins (CCPs). Dysregulated expressions or functional impairment of these regulators may result in unchecked complement activity, contributing to platelet dysfunction and heightened thrombo-inflammatory responses [58].

Additional complications from complement activation include the C5a-mediated signaling of C5aR1 and C5aR2 in neutrophils. The activation of these pathways promotes neutrophilic infiltration and inflammatory responses, promoting cardiopulmonary complications such as atherosclerosis [58]. Activation of C3a and C5a mediated pathways on vascular endothelium has also been shown to promote vascular EC migration and modulation of B & T cell activation, leading to further vessel wall remodeling [59]. Notably, in atherosclerosis murine models, C5aR signaling was also shown to promote angiotensin II-mediated cardiac hypertrophy and remodeling, linking complement activation to downstream CVDs [59].

## 3. Platelets and Plaque Instability

### 3.1. Mechanisms of Plaque Destabilization

**Figure 3:**
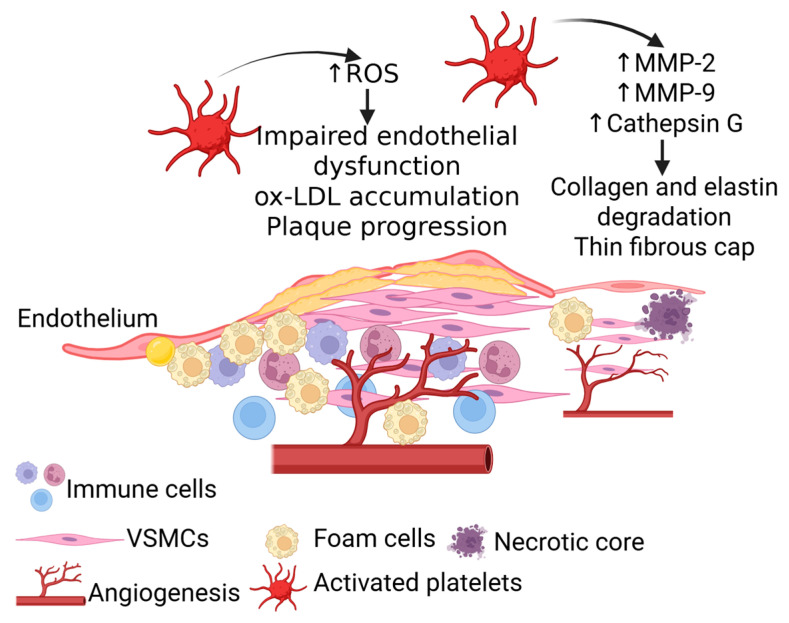
Platelets and plaque rupture. Increased reactive oxygen species (ROS) contribute to endothelial dysfunction, oxidized low density lipoprotein (ox-LDL) accumulation, and immune cell recruitment leading to plaque progression. Further, increased matrix metalloproteinases (MMPs) contribute to collagen and elastin degradation resulting in thin fibrous cap. Persistent inflammation and the presence of necrotic core in the plaque results in plaque rupture. ↑ indicates increased expression.

Plaque destabilization reflects the convergence of cellular, enzymatic, and oxidative processes that progressively weaken the fibrous cap. VSMCsV, normally residing in the medial layer, are recruited to the intima in response to PDGF, cytokines, and chemoattractants such as TNF-α [60]. Once in the intima, these cells undergo a phenotypic switch from contractile to synthetic, secreting ECM proteins that initially provide structural reinforcement to the fibrous cap [60,61]. However, sustained inflammatory signaling and oxidative stress gradually undermine this stabilizing effect, leading to cap thinning and an increased susceptibility to rupture [60]. Platelets amplify this destabilization through the secretion of proteolytic enzymes, including matrix metalloproteinases (MMP-2, MMP-9) and cathepsin G, which degrade collagen and elastin within the ECM [10,31] (Figure 3). This enzymatic activity not only compromises the cap’s strength but also promotes the development of a necrotic core, a hallmark of advanced lesions (Figure 3). The accumulation of necrotic material beneath the plaque surface further undermines stability, and eventually, exposure of this core establishes a potent trigger for thrombosis [10].

A parallel contribution comes from ROS. Platelet-derived ROS intensify oxidative stress within the lesion, impairing endothelial function, driving lipid oxidation, foam cell formation, and promoting ox-LDL accumulation [60]. Elevated ROS perpetuates a cycle of self-amplification, termed ROS-induced ROS release, that accelerates plaque progression. Activated platelets, by agonists like collagen or thrombin, produce ROS, which in turn causes further platelet activation and damage to the vessel wall significantly contributing to the development and progression of atherosclerosis by promoting inflammation, platelet activation, and thrombosis [60,62] (Figure 3). This redox imbalance encourages foam cell formation and necrotic core expansion, while comorbid conditions such as hypertension and diabetes exacerbate ROS-mediated platelet activation, increasing the likelihood of plaque rupture, arterial thrombosis, and cardiovascular events [63,64] (Figure 3). Adding further complexity, platelet–neutrophil interactions serve as an additional destabilizing force. These interactions enhance local inflammation, stimulate protease release, and erode the structural integrity of the plaque. This leaves atherosclerotic plaques vulnerable to rupture, leading to thrombotic events such as MI or stroke [10].

### 3.2. Plaque Rupture and Thrombosis

Once the fibrous cap ruptures, circulating platelets rapidly adhere to the exposed subendothelial matrix through glycoprotein receptors that bind collagen and vWF. This adhesion initiates platelet activation and aggregation, a process further amplified by the release of ADP, thromboxane A_2_, and serotonin [10]. Fibrin deposition leads to the development of microthrombi within the plaque core, further contributing to instability [10,65]. In parallel, activated neutrophils infiltrate the rupture site and release NETs, which provide a prothrombotic scaffold that enhances platelet adhesion and exacerbates endothelial dysfunction. As atherosclerosis advances chronically, platelets shift toward a hyperactive state. They become more responsive to agonists such as ADP and thrombin, and demonstrate increased calcium mobilization and cytoskeletal rearrangement, changes that not only sustain prothrombotic activity but also contribute to lesion calcification [10,35]. Localized hypoxic conditions promote erythrocyte and platelet destruction and stimulate neovascularization of the vasa vasorum from the adventitia into the intima, a change that increases the risk of intraplaque hemorrhage [10,36]. Collectively, these mechanisms drive plaque progression toward rupture and the development of major adverse cardiovascular events such as acute coronary syndromes (ACSs) and cardiovascular death [10].

### 3.3. Inflammation-Driven Rupture Risk

Inflammatory processes are tightly interwoven with platelet activation, creating a feedback loop that magnifies plaque vulnerability. Activated platelets engage neutrophils through receptor–ligand interactions or soluble mediators, triggering the release of NETs. The negatively charged DNA and histones within NETs provide a potent procoagulant surface, enhancing platelet adhesion, amplifying local inflammation, and sustaining thrombin generation [66]. Moreover, complement activation, particularly via the C5a pathway, can further promote NETosis through signal transducer and activator of transcription 3 (STAT3) and mitochondrial ROS-dependent mechanisms. Clinically, elevated NET markers correlate with infarct size, recurrence of stroke, hypercoagulability, and poor outcomes in patients with MI, underscoring their pathogenic relevance [67,68,69,70,71].

In parallel with NET-driven pathways, platelets contribute directly to endothelial dysfunction. Through the accumulation of ROS, platelets inactivate NO, disrupting endothelial-mediated vasodilation and leading to increased vascular stiffness and contractility [72]. This imbalance heightens susceptibility to plaque rupture and thrombosis. Platelet secretion of proinflammatory mediators such as TNFα can also induce endothelial apoptosis or necrosis, while surviving ECs shed their glycocalyx (GCX) [72,73]. GCX, a sugar-rich protective barrier that normally regulates vascular tone and limits platelet and leukocyte adhesion, becomes degraded under pathological shear stress and inflammatory conditions. Loss of this barrier unmasks adhesion molecules, promotes neutrophil and platelet capture, and permits LDL infiltration into the subendothelial space [74]. The consequences of GCX degradation are profound. LDL particles aggregate beneath the endothelium, attracting monocytes that differentiate into macrophages and eventually die, contributing to necrotic core expansion. As the necrotic core grows, the structural integrity of the plaque is progressively undermined [74]. Once rupture occurs, exposure of this highly thrombogenic material to circulating blood elements initiates rapid platelet accumulation, reinforcing thrombus formation [31]. Together, NETosis, endothelial dysfunction, and GCX degradation exemplify how platelet-driven inflammatory pathways establish a prothrombotic environment that significantly elevates rupture risk.

## 4. Therapeutic Intervention

### 4.1. Current Antiplatelet Strategies

**Figure 4:**
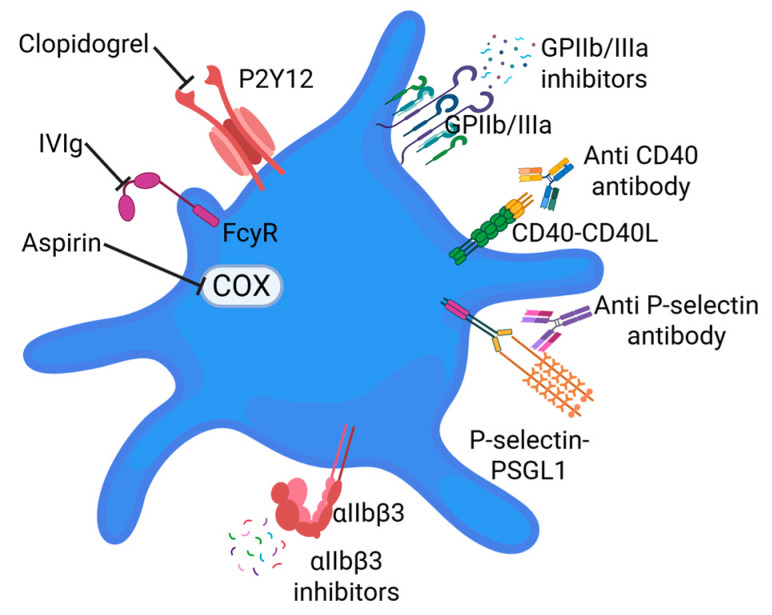
Targeting platelets to attenuate plaque progression and rupture. Inhibiting platelet activation, the interaction between platelets and immune cells, and platelet aggregation are potential strategies to attenuate platelet progression and rupture thereby reducing acute ischemic events.

Antiplatelet therapeutic strategies serve as key preventative and mitigation measures of cardiovascular events, particularly in patients with ACS and atherosclerosis. Aspirin, P2Y12 inhibitors (e.g., clopidogrel), and GPIIb/IIIa (inhibiting platelet aggregation) represent a wide variety of strategies to target specific mechanisms in platelet activation and aggregation (Figure 4). However, there are some challenges like bleeding risks and variability in patient response (clopidogrel resistance). This resistance increases the risk of cardiovascular events, such as heart attack and stroke, and is linked to factors like genetic variations in the CYP2C19 enzyme, certain co-occurring conditions like hypercholesterolemia, and sometimes even patient-specific factors such as ethnicity and age. In atherosclerosis, higher platelet reactivity due to resistance is associated with more advanced disease and a greater risk of complications after interventions like stenting [75]. Lack of biomarkers to guide therapy is another concern. However, recent studies suggest that integration of specific biomarkers, genotype and phenotype-related data in antiplatelet therapy stratification in patients will be of great clinical significance and more effective, personalized therapy [76]. A study by Kadoglou et al. [77] suggested that novel imaging and biochemical biomarkers are positively associated with atherosclerosis severity and may play a significant role in identifying vulnerable plaques.

Aspirin, extensively used for its antiplatelet properties, irreversibly inhibits cyclooxygenase-1 (COX-1) [78]. Huseynov et al. [24] expand upon thrombin’s enzymatic development in the coagulation cascade, which serves to activate human platelets through receptors PAR1 and PAR4 and ensures platelet response to vascular injury. The coagulation cascade progresses as dense granules release the ADP that stimulates P2Y1 and P2Y12 receptors, and the thromboxane derived from COX-1 stimulates the thromboxane prostanoid receptor [24]. Aspirin’s inhibition of COX-1 suppresses thromboxane A2, therefore reducing thromboxane synthesis and lowering cardiovascular risk [78].

Kessler et al. [78] compare recent findings of current platelet treatment, detailing P2Y12 and GPαIIb/IIIa receptors as targeted mitigation strategies in some patients after coronary stenting or in ACS. P2Y1 and P2Y12 receptors coupled to different G proteins serve different functions; P2Y1 initiates shape change in platelets and induces platelet aggregation, while P2Y12- a clinically experimented antiplatelet drug- serves as an antagonist, inhibiting platelet aggregation [35]. In the clinical studies involving patients with CAD, antiplatelet drugs clopidogrel, prasugrel, and ticagrelor target P2Y12 to reduce thrombosis risk. These therapeutics work to inhibit the release of EVs from platelets, known to cause inflammation and thrombosis. Additionally, animal studies show the P2Y12 receptor’s participation in atherogenesis by enhancing the proliferation of VSMCs and endothelial dysfunction [35]. Therefore, the deletion of the P2Y12 gene in the mouse model resulted in reduced lesion size, decreased macrophage infiltration, and increased fiber within the plaque. The results from this comparative investigation indicate that the early onset of atherosclerosis is mainly linked to the expression of P2Y12 in the blood vessel’s wall, rather than the platelet [35]. The anti-inflammatory properties of antiplatelet drugs are conveyed as possible therapeutics in the search for primary prevention against atherosclerosis.

Following vascular injury, activated platelets express αIIbβ3 integrin. The activated integrin forms a bridge to fibrin(ogen) or vWF and subsequently links platelets together to plug the site of vascular trauma. While integrin aIIbβ3 is understood to mediate hemostatic and thrombotic function, its role in the promotion of platelet activation can be explored to bridge gaps in atherosclerotic translational research. Wang et al. emphasize proinflammatory effects of integrin αIIbβ3, deducing macrophage deletion or transplantation of β3 aggravated atherosclerotic lesions [35]. Platelet αIIbβ3 displays contradictory effects as inhibitors of integrin αIIbβ3 (e.g., abciximab, tirofiban, and eptifibatide) can treat unstable, ischemic events, while the complete absence of integrin β3 in atherosclerosis-prone mice enhances vulnerability to inflammation and lesions (Figure 4). In αIIbβ3 deficient murine models, its paradoxical, context-dependent roles are elucidated as the partial loss of αIIbβ3 impairs platelet adhesion and aggregation, while αIIbβ3 deficient platelets express a reduction in anti-inflammatory mediators, destabilizing atherosclerotic lesions contingently [79]. The contradictory roles of integrin αIIbβ3 highlights the importance of platelet interactions with ECs and leukocytes, beyond its hemostatic functions. Further research to specify the targeted role of αIIbβ3 in atherosclerosis is recommended to be achieved through platelet-specific knockout β3 [35].

### 4.2. Emerging Therapeutic Targets

Extending beyond conventional methods of targeting mechanisms, newer strategies center on the modulation of platelet-driven inflammation and vascular interactions. The disruption of platelet–endothelial interactions, by blocking the P-selectin/PSGL-1 axis (Figure 4), is an emerging antiplatelet therapeutic strategy. Kessler et al. [78] provide insight into P-selectin as a key player in the activation of adhesion receptors, initiating platelet aggregation with leukocytes and ECs after vascular injury. The role of P-selectin is significant in platelet adhesion on ECs) as well as leukocyte tethering and transmigration towards the site of inflammation. Seen in bone marrow transplantation models, enhanced P-selectin expressed on ECs (ECs) and platelets worsened atherosclerosis [78]. Conceptually, the inhibition of the PSGL-1 axis would result in a decrease in endothelial activation and the progression of inflammatory mechanisms. However, Kessler et al. [78] assert that randomized controlled trials monitoring plaque development would be warranted to draw conclusive beneficial effects of blocking P-selectin on atherogenesis in humans.

A strategic therapeutic alternative is the modulation of platelet-secreted alarmins, such as high mobility group box protein 1 (HMGB1) and Cyclophilin A. HMGB1 and Cyclophilin A are molecules that alarm and amplify immune responses, playing significant roles in endothelial dysfunction, leukocyte recruitment, and NETosis [78]. Platelet microparticles in chronic inflammation are fostered by their interaction with HMGB1, triggering neutrophils to cast their NETs. Wang et al. [35] detail how reduced atherosclerotic development is due to the therapeutic blockade of HMGB1; this corroborates Kessler’s [78] understanding of platelet-derived HMGB1’s effects in NET formation and atherosclerotic thrombosis. Cyclophilin A (CyPA) proteins are released during platelet activation and are located within atherosclerotic plaques [35]. While widely understood as a protein that stimulates and proliferates VSMCs, expresses adhesion molecules in ECs, and promotes atherosclerosis, CyPA is an emerging platelet-derived mediator that requires in-depth research to determine its role in plaque formation. Conclusively, the neutralization of these alarmins attenuates inflammation and mitigates tissue degeneration in thromboinflammatory conditions.

### 4.3. Modulating Platelet–Immune Crosstalk

Promising evidence signifies the essential function platelet–immune interactions play in atherothrombosis and vascular inflammation. Wang clarifies the inflammatory mediation of platelets by defining platelet interaction with ECs and leukocytes to be key participants in atherosclerotic lesion formation [35]. Platelet monocyte interactions influence endothelial dysfunction and thromboinflammatory cascading as complement-activated platelets engage monocytes via immune signaling. Consisting of plasmatic proteins that contribute to immune response and homeostasis processes like angiogenesis, tissue regeneration, and apoptotic cell clearance, the complement system serves as an important part of the immune system, yielding both pro- and anti-atherogenic effects [37]. Nording et al. expands on the complement system’s interaction with platelets through the release of complement factors and regulatory proteins, C3a and soluble membrane attack complex (sMAC) [37].

Upon vascular trauma and its subsequent activation, platelets express CD40 ligand (CD40-L) [37]. In early-stage plaque development, CD40L serves as a proinflammatory cytokine rapidly released to the membrane surface after platelet activation [35]. CD40L interacts with CD40 on monocytes and ECs to promote tissue factor expression, cytokine release, and leukocyte recruitment towards the injury site. Circulating soluble CD40-L (sCD40L) amplifies plaque vulnerability in highly activated regions while supporting immune responses and tissue repair in less inflamed environments [35]. Because platelets serve as the main source of sCD40L, this suggests sCD40L to be a profound indicator of postoperative risk of cardiovascular disease. Moreover, consistently higher sCD40L levels in atherosclerotic patients highlight the possibility of this inflammatory mediator becoming a noninvasive method for atherosclerosis detection [74]. In atherosclerotic-prone mice, disrupting CD40 signal transduction with CD40L antibodies (Figure 4) or CD40L deficiency was determined to improve atherosclerotic lesions by reducing macrophage infiltration [35]. However, clinical studies, showed that the lack of CD40L signaling axes demonstrated an instability of vascular immune regulation [80]. The adverse effect expressed when interfering with sCD40L signal transduction in CAD patients is likely due to the unsafe exposure to long-term inhibition of inflammatory mediators, severely increasing thromboembolic risk. The role of CD40 in atherosclerotic progression yields contradictory results, because its proatherogenic or anti-inflammatory effects are dependent on the stage of plaque progression, involvement of specific cell types, localized inflammation, and therapeutic targeting specificity. To clarify the significance of targeted intervention, specific inhibition of circulating sCD40L may preserve its immunoregulatory effects, while avoiding thrombotic risks and immune suppression associated with the inhibition of membrane-bound CD40L. The disputable findings regarding the roles of context-dependent CD40 and CD40L underscore their importance in vascular activation and why they would serve as compelling therapeutic targets in CVD management.

### 4.4. Platelet–Complement System Interventions

Platelets engage with special pathways of the complement system by secreting complement and regulatory components. Nording et al. [37] reported that elevated levels of C3a and sMAC are linked to amplified cardiovascular risk, and expression of platelet C3aR/C5aR1is directly linked to platelet activation in CAD patients, thus concluding the platelet interaction to play a causal factor in atherosclerosis. By inhibiting C5aR1, the receptor antagonists yield antiatherogenic effects in atherosclerotic animal models. C3a/C5a receptor antagonists aim to selectively target the inflammation from complement activation while sustaining their natural mechanisms [37]. Evidently, the clinical use of the complement-targeted drug, eculizumab, and the notoriety emerging from its successful clinical use have been discussed. Eculizumab is an anti-C5 antibody serving as a treatment for complement-mediated conditions sharing hemolytic and thrombotic processes [37]. Though there are limited studies assessing the efficacy of eculizumab in clinical trials of atherosclerosis, the drug inhibits formation of the terminal complement complex and has been shown to prevent thrombus formation, consistent with its anticoagulative and antiplatelet activity [81]. Preclinical trials conducted by Golomingi et al. [82] have also shown promise in establishing the importance of complement inhibition in preventing platelet activation and thrombosis. In this trial, they mimicked human vasculature by using a microvascular bleeding model, lined with ECs and perfused with human whole blood to assess platelet–complement interactions upon mechanical injury. Interestingly, they found that C3 and C5 inhibitors were associated with significant reduction in fibrin formation and that C3 inhibitors specifically were associated with reduced platelet activation. Additionally, triple inhibition of all 3 complement pathways via a novel triple fusion inhibitor (TriFu) reduced both fibrin formation and platelet activation and had the strongest effects on clot formation. Specific complement inhibitors that target platelets to reduce vascular inflammation and plaque progression highlight the clinical possibilities of complement-based therapies to prevent cardiovascular events.

### 4.5. Nanotechnology and Precision Drug Delivery

Invasive endarterectomies and long-term medications, like aspirin, aiding in cholesterol management often come with complications that increase the risk of ischemic events like MI or limited arterial dilation [38,83]. Inspired by the platelets’ natural ability to bind to plaques, a noninvasive, biomimetic photodynamic therapeutic strategy was designed to intervene in the progression of atherosclerotic plaques. While treatment for atherosclerotic progression entails lifestyle modifications centering physical activity, a heart-healthy diet, and smoking cessation, photodynamic therapy (PDT) works to improve CVD management mechanistically [84].

Using the body’s delivery mechanism, upconversion nanoparticles (UCNPs) are loaded with a light-sensitive drug, chlorin e6 (Ce6), to target the foam cell formation stage of plaque aggregation. PDT utilizes autophagy to induce cell death and regulate the lipid-rich core in macrophage-derived foam cells [38]. To lessen the limitation of the photosensitizer’s ability of only being activated by visible light, UCNPs convert near-infrared photons to visible wavelengths, allowing deep tissue penetration in vivo. The UNCPs’ penetration deep into the body allows the near-infrared light to target the plaque’s core, absorb it, and eternally re-emit as visible red light [38]. The visible red light activates the C6 photosensitizer, causing the production of ROS to preferentially target and treat foam cells, while limiting harm to any surrounding healthy tissue [38]. The biomimetic nanoparticles interact with the inflamed endothelium and activate cells by binding to the thrombotic sites of inflammation. By delivering antithrombotic agents directly to the lesions, PDT emphasizes precision targeting to avoid excessive bleeding risk.

To ensure PDT re-emits light at its target in vivo, the platelet membrane-coated nanoparticle labeled with indocyanine green (Icg) is visually tracked with a high-resolution camera [38]. The functional accumulation of radioactivity in deep tissue is visualized with a labeled radioactive marker. The atherosclerotic murine model used in this study presented fluorescent signals in the left carotid artery where plaques were induced; this showed precise localization of plaques in vivo [38]. This selective cytotoxicity offers promising modulation strategies in preclinical studies by depicting a reduction of M4 macrophage density within plaques and improved plaque stabilization [84]. Analytical detection of nanoparticle accumulation in the lipid core plaque, where foam cells reside, were visualized through hematoxylin and eosin (H&E) staining [38]. Guided laser radiation in PDT continues to serve as a revolutionary therapeutic strategy in targeted plaque treatment, intentionally avoiding degeneration of healthy tissues [38].

### 4.6. Clinical Limitations and Translational Novelty

Despite compelling preclinical efforts to mitigate the progression of atherosclerosis, several limitations hinder the clinical translation of complement inhibition, nanoparticle delivery, and immunomodulation. While in atherosclerotic murine models, the C3a/C5aR1 complement inhibition pathway initially attenuates atherogenesis by limiting vascular inflammation and plaque development, this inhibitory system could inversely disrupt hemostasis and dampen immune response. Clinical application could compromise the patient’s immune defense to infections and impair wound healing. Patient variability is a recurring limitation arising as a response to antiplatelet strategies. This is exemplified by clopidogrel which serves as a P2Y12 inhibitor [35]. Because the reactivity of platelets may be altered unpredictably, genetic variability complicates the feasibility of integrating platelet-modulatory therapies into conventional treatments. Under infrared light, UCNPs have the therapeutic ability to precisely image and target drug delivery noninvasively. However, ensuring efficient target optimization, minimizing off-target effects, and ensuring long term biocompatibility act as limiting factors to this emerging method [85].

Though GPIIb/IIIa inhibitors have displayed antithrombotic effects, they pose significant bleeding risks in patients with coexisting conditions or undergoing invasive procedures. Additionally, because the therapeutic window for GPIIb/IIIa is narrow and necessitates intravenous administration, this further limits the benefits of this therapeutic strategy intended to aid in chronic atherosclerotic management [86]. Similarly, though CD40/CD40L modulatory therapies have shown efficacy in impairing proinflammatory signaling and weakening plaque vulnerability, the broad role of CD40 in platelet activation and immune mediation presents an increased risk of thromboembolic events when altered with limited specificity. Conversely, the CD40/CD40L signaling pathway, affecting innate and adaptive immunity, offers an ideal opportunity to selectively modify proteins without compromising systemic immune response. Tumor necrosis factor receptor-associated factors (TRAFs) are involved in immune regulation, and selectively modulating its axis with receptor CD40 in macrophages has demonstrated reduction in macrophage infiltration and plaque burden, without risking thromboembolic events [87]. Translational studies are currently exploring the clinical application of the CD40-TRAF6 axis, carefully considering the hyper-specificity of this treatment and genetic variability of patients with comorbidities.

P-selectin, found on activated platelets and endothelial cells, binds to PSGL-1 which is expressed on leukocytes like monocytes and dendritic cells. This interaction promotes thrombosis and initiates an inflammatory cascade, the key processes deriving atherosclerosis. Dendritic cell maturation and activation via the TLR4/NF-κB signaling pathway contribute to the development and progression of atherosclerosis. Blockading this interaction with antibodies or other antagonists has been shown in animal models to reduce atherosclerotic lesions and related complications [88,89]. P-selectin/PSGL-1 blockade shows promise in treating atherosclerosis by inhibiting inflammatory cell recruitment to arterial plaques. Antagonism of P-selectin may ameliorate accumulation of macrophages in the allograft during antibody-mediated rejection [90]. Targeting the CXCL4–CCL5 heterodimer is a promising strategy for treating atherosclerosis by inhibiting the recruitment of inflammatory monocytes to arterial walls. The interaction between these two chemokines, released by activated platelets, amplifies the inflammatory response that drives the formation of atherosclerotic plaques [91,92].

### 4.7. Clinical Trials Associated with Platelets

Clinical trials for antiplatelet therapy in atherosclerosis focus on improving outcomes and reducing risks by evaluating new agents and strategies. Key areas of research include assessing dual antiplatelet therapy (DAPT) versus single therapy with agents like aspirin or clopidogrel, particularly in acute events like stroke or following stenting, while managing bleeding risks. Newer trials explore different drugs, timeframes, and combinations, such as the addition of new antithrombotics like rivaroxaban to aspirin, aiming for better personalized treatment for patients with atherosclerotic disease. Intensive Statin and Antiplatelet Therapy for Acute High-Risk Intracranial or Extracranial Atherosclerosis (INSPIRES) trial evaluated DAPT with clopidogrel and aspirin for mild ischemic stroke or high-risk TIA, showing it reduced recurrent stroke risk compared to aspirin monotherapy within 72 h of symptom onset. Antiplatelet Therapy in Acute Mild–Moderate Ischemic Stroke (ATAMIS) trial was one of the first large trials to demonstrate that DAPT with clopidogrel and aspirin reduced the risk of early neurological deterioration in patients with acute mild to moderate ischemic stroke within 48 h. COMPASS trial assessed the benefit of adding rivaroxaban to aspirin for patients with chronic coronary artery disease. CAPRIE study established the importance of newer antiplatelet agents, comparing clopidogrel and aspirin in patients with various atherosclerotic diseases [93,94].

## 5. Conclusions, Challenges and Future Directions

Platelets act as multifaceted contributors to atherogenesis and ischemic events, playing active roles in vascular inflammation and endothelial dysfunction, and emerging as the central orchestrators of the thrombo-inflammatory programs that drive atherosclerosis [35]. As atherosclerotic lesions form, immune cell recruitment, foam cell formation, complement activation, plaque destabilization, platelet adhesion receptors, granular chemokines (CXCL4, CCL5), cytokines (IL-1β, TNF-α), and crosstalk with neutrophils and complement systems create an integrated network tightly coupling thrombosis to vascular inflammation [13,14,15,16]. Understanding this mechanism allows for a fundamental shift in therapeutic approaches to cardiovascular prevention. Considering the prevalence of atherosclerosis and its relation to various cardiovascular diseases, a comprehensive understanding will allow for more effective modulation of platelet activity, targeting not only aggregation pathways but also inflammatory signaling cascades. COX-1 and P2Y12 inhibition remain cornerstones of secondary prevention, effectively disrupting amplification loops involving ADP and thromboxane A2. However, despite these avenues of antiplatelet therapy, persistent residual cardiovascular risk, combined with inherent bleeding constraints, highlights the need for novel approaches that can target platelet-driven inflammatory processes while preserving essential hemostatic function.

Several promising therapeutic avenues directly address this gap by targeting key platelet–inflammatory interfaces. Blocking platelet–endothelial and platelet–leukocyte interactions through inhibition of the P-selectin/PSGL-1 axis offers a potential strategy to reduce EC activation and subsequent inflammatory cascades. Additionally, disrupting the CXCL4-CCL5 heterodimer formation offers a potential path for decreasing the recruitment of monocytes and the formation of plaques and NETs. Furthermore, altering the C3a/C3aR axis aims to selectively decrease inflammation-induced complement activation while preserving the pathways’ immune function. In addition, C5aR1 antagonists have shown promising antiatherogenic effects in murine models and should be further explored in human models. However, clinical trials with Eculizumab are also being conducted and offer a potential avenue for more research as well. Nanoparticles also offer a novel therapeutic opportunity to disrupt foam cell development and plaque formation. These targets collectively aim to preserve physiological hemostatic capacity while suppressing pathological inflammatory activation that drives plaque progression and instability.

Despite the efficiency of antiplatelet therapeutic agents, they present a well-known risk of bleeding. Nanoparticle-derived systems are more than capable of releasing drugs at localized sites; however, adverse effects, immunogenicity, and long-term safety must be investigated further. Because PDT is an emerging technique targeting foam cells within plaques, the potential for inadvertent activation of immune cells has yet to be fully elucidated. While bio-mimetic nanoparticles, complement inhibitors, and alarmin-inhibiting agents have demonstrated preclinical promise in murine models and in vitro systems, translating these findings to human studies continues to pose a significant obstacle. The reproducibility of nanoparticle synthesis and limited data regarding the efficacy in different patient populations are a few limitations that pose challenges when bridging preclinical studies with clinical application. When bridging the gap, interdisciplinary collaboration between immunologists, scientists, and specialized doctors is key.

Biomarkers like proteins, mRNAs, or genetic mutations can predict the risk of disease, guide the dosage of selected medicine, and monitor therapeutic and adverse responses. While future studies seek to identify medicinal blockers of CD40L-mediated platelet interactions with inflammation, current antiplatelet targeting therapies circulate sCD40L, PEVs, and complement fragments to provide a basis for personalized treatment [35]. Additionally, PDT serves as exemplary biomarker-guided therapy by activating targeted sites and directing photosensitizers to foam cells in atherosclerotic plaques.

Despite the relatively uniform pathophysiology of atherosclerosis, diverse patient populations exhibit variable drug responses, resistance, and susceptibility to downstream complications [95]. For example, genetic polymorphisms affecting P2Y12 receptor sensitivity, COX-1 activity, or CD40L expression can alter individual responses to antiplatelet agents. Similarly, elevated circulating biomarkers such as sCD40L, CXCL4, or platelet-derived extracellular vesicles may predict thrombotic risk and guide the intensity of antiplatelet therapy. Clinical studies have also shown that personalized treatments can reduce side effects, improve treatment efficacy, and minimize potential risks [95].

Platelets host a broad range of roles in inflammation and immune response, solidifying them as key targets for therapeutic approaches. Biomarkers like sCD40L, PEVs, and complement fragments allow for precision-guided interventions. Eculizumab selectively blocks the complement system, while PDT uses light-activated nanoparticles to selectively treat plaque-derived foam cells. Despite these technological and immunomodulatory advances, immune responses and long-term risk pose safety concerns. Integrating clinical relevance into these successful experiments will demand interdisciplinary coordination and patient-oriented solutions.

## Data Availability

No new data were created or analyzed in this study.

## Abbreviations

The following abbreviations are used in this manuscript:

ACS	Acute coronary syndromes
CAD	Coronary artery disease
CCL5	C-C motif ligand 5
CCR1	C-C chemokine receptor type 1
COX1	Cyclooxygenase-1
CVD	Cardiovascular disease
CXCL1	C-X-C motif chemokine ligand 1
CXCL4	C-X-C motif chemokine ligand 4
ECM	Extracellular matrix
ECs	Endothelial cells
EVs	Extracellular vesicles
GCX	Glycocalyx
HMGB1	High mobility group box protein 1
IL	Interleukin
ICAM1	Intercellular Adhesion Molecule 1
MI	Myocardial infarction
MCP1	Monocyte chemoattractant protein-1
MAC	Membrane attack complexes
MMPs	Matrix metalloproteinases
NETs	Neutrophil-extracellular traps
NO	Nitric oxide
NPA	Neutrophil–platelet aggregates
ox-LDL	oxidized low-density lipoprotein
PAD	Peripheral artery disease
PDT	Photodynamic therapy
PF4	Platelet factor 4
PMC	Platelet–monocyte complexes
PMPs	Platelet microparticles
PDGF	Platelet-derived growth factor
PSGL1	P-selectin glycoprotein ligand-1
RANTES	Regulated upon activation normal T expressed and secreted
ROS	Reactive oxygen species
STAT3	Signal Transducer and Activator of Transcription 3
TNF-α	Tumor necrosis factor-alpha
TGF-β	transforming growth factor beta
UCNPs	Upconversion nanoparticles
VCAM1	Vascular Cell Adhesion Molecule-1
VEGF	Vascular endothelial growth factor
VSMCs	Vascular smooth muscle cells
vWF	von Willebrand factor
